# An Internet-Based Method for Extracting Nursing Home Resident Sedative Medication Data From Pharmacy Packing Systems: Descriptive Evaluation

**DOI:** 10.2196/jmir.6938

**Published:** 2017-08-03

**Authors:** Tristan Ling, Peter Gee, Juanita Westbury, Ivan Bindoff, Gregory Peterson

**Affiliations:** ^1^ Unit for Medication Outcomes Research and Education Division of Pharmacy, School of Medicine University of Tasmania Hobart Australia; ^2^ Wicking Dementia Research and Education Centre Faculty of Health University of Tasmania Hobart Australia

**Keywords:** electronic health records, information storage and retrieval, inappropriate prescribing, antipsychotic agents, benzodiazepines, nursing homes, systematized nomenclature of medicine, health information systems

## Abstract

**Background:**

Inappropriate use of sedating medication has been reported in nursing homes for several decades. The Reducing Use of Sedatives (RedUSe) project was designed to address this issue through a combination of audit, feedback, staff education, and medication review. The project significantly reduced sedative use in a controlled trial of 25 Tasmanian nursing homes. To expand the project to 150 nursing homes across Australia, an improved and scalable method of data collection was required. This paper describes and evaluates a method for remotely extracting, transforming, and validating electronic resident and medication data from community pharmacies supplying medications to nursing homes.

**Objective:**

The aim of this study was to develop and evaluate an electronic method for extracting and enriching data on psychotropic medication use in nursing homes, on a national scale.

**Methods:**

An application uploaded resident details and medication data from computerized medication packing systems in the pharmacies supplying participating nursing homes. The server converted medication codes used by the packing systems to Australian Medicines Terminology coding and subsequently to Anatomical Therapeutic Chemical (ATC) codes for grouping. Medications of interest, in this case antipsychotics and benzodiazepines, were automatically identified and quantified during the upload. This data was then validated on the Web by project staff and a “champion nurse” at the participating home.

**Results:**

Of participating nursing homes, 94.6% (142/150) had resident and medication records uploaded. Facilitating an upload for one pharmacy took an average of 15 min. A total of 17,722 resident profiles were extracted, representing 95.6% (17,722/18,537) of the homes’ residents. For these, 546,535 medication records were extracted, of which, 28,053 were identified as antipsychotics or benzodiazepines. Of these, 8.17% (2291/28,053) were modified during validation and verification stages, and 4.75% (1398/29,451) were added. The champion nurse required a mean of 33 min website interaction to verify data, compared with 60 min for manual data entry.

**Conclusions:**

The results show that the electronic data collection process is accurate: 95.25% (28,053/29,451) of sedative medications being taken by residents were identified and, of those, 91.83% (25,762/28,053) were correct without any manual intervention. The process worked effectively for nearly all homes. Although the pharmacy packing systems contain some invalid patient records, and data is sometimes incorrectly recorded, validation steps can overcome these problems and provide sufficiently accurate data for the purposes of reporting medication use in individual nursing homes.

## Introduction

### Reducing Use of Sedatives: “RedUSe”

It has been shown that residents within nursing homes often receive sedating medications contrary to guidelines [1-6]. A multi-strategic, interdisciplinary intervention called Reducing Use of Sedatives (RedUSe) was developed in Tasmania in 2008 aiming to address inappropriate sedative use in nursing homes [7], where the term “sedative” referred to “psycholeptic” medication or antipsychotic or anxiolytic or hypnotic classes (see Table 1 in the Methods section of this paper for the Anatomical Therapeutic Chemical (ATC) codes for included medications). The project approached the problem of inappropriate sedative use by

1. Performing an audit of sedative use across all residents in the nursing home.
2. Presenting audit feedback to nursing home staff, benchmarked against average nursing home sedative use, during an interactive education session.
3. Developing personalized sedative review plans for each resident taking sedative medication, with input from a “champion nurse” and the home’s pharmacist, for the attention of the prescriber.

All steps were repeated 3 months after the initial audit, and steps 1 and 2 repeated again at 6 months.

The RedUSe program was tested in a controlled trial in 2009 with 25 homes [7]. Most nursing homes obtain their medications from community pharmacies that utilize commercial computerized medication packing systems to record and pack each resident’s medications into separate blister packs or sachets. Audit data was collected by installing software in each supply pharmacy to extract residents’ medications from these dispensing and packing databases. The software was compatible with the two most common dispensing and packing systems in Australia, FRED [8] and Webstercare [9]. Data mappings were created between these two systems’ antipsychotic and benzodiazepine identifiers to ATC codes, allowing automated production of audit reports charting the prevalence of use of each of these drug groups in each nursing home. This process required an in-person visit to the supply pharmacy and the nursing home for verification against resident medication charts [7].

The RedUSe trial significantly reduced rates of both antipsychotic and benzodiazepine use in intervention homes when compared with control homes [7]. This success led to funding from the Australian government to expand the project on a national scale. However, the remoteness of some nursing homes and the scale of data collection necessitated an improved data extraction method.

### Extracting Nursing Home Medication Data

A recent systematic review identified 22 interventions that have been developed to address inappropriate antipsychotic use in nursing homes [4]. This review noted that medication audit initiatives typically collect data by visiting the home and copying resident charts [4,10,11] using cohorts already detailed by other studies [12,13] or by accessing electronic pharmacy records [14,15].

Electronic extraction of medication records from the nursing homes themselves was not possible, as at the time of project implementation many nursing homes were not using electronic medication records. Similarly, the relatively new Australian national electronic health record (EHR) system (formerly the Personally Controlled Electronic Health Record, now My Health) still has a low adoption rate, both from health practitioners and patients [16]. Although prescriptions are being increasingly computerized at the point of prescription, they are not universally so, and accessing the computer systems of each prescriber for each resident in a nursing home would be impractical.

Community pharmacy records are an increasingly viable source of data [17]. Many examples of data collection using pharmacy records exist, with outcomes including an influenza monitoring system [18], validating hospital admission drug charts [19], providing decision support to pharmacists [20], quantifying medicine use [21], and identification of patients for intervention [22,23]. Pharmacies are also a condensed source, as it is generally expected that a nursing home is supplied medications by one or two pharmacies. Additionally, pharmacies often supply multiple nearby nursing homes. Literature has noted that, thus far, pharmacy data collection procedures have typically been small scale, performed by one individual, and lacking a procedure for checking accuracy [4], providing sufficiently accurate data for aggregate analysis but not for personalised intervention.

For larger scale studies, the remoteness of some nursing homes makes in-person visits impractical. An alternative is to recruit a person already employed by each nursing home: within this project a “champion nurse” was designated within each home to promote and lead the project, as published literature has consistently noted this as critical to success [24-28]. However, other project requirements left little capacity for the champion nurse to also facilitate data collection; considering it has been reported that the three most stressful factors in aged care nursing are “not having enough staff,” “having too much work to do,” and “interruptions to regular work” [29], performing significant data entry in addition to promoting cultural change is likely too large a burden.

### Using Extracted Medication Data

There are a small number of pharmacy medication packing systems in use in Australia, yet each store their data in very different formats. To be able to identify medications of interest and report on them meaningfully, an extract, transform, and load (ETL) process is required that can translate medicines to a common standard for comparison.

**Figure 1:**
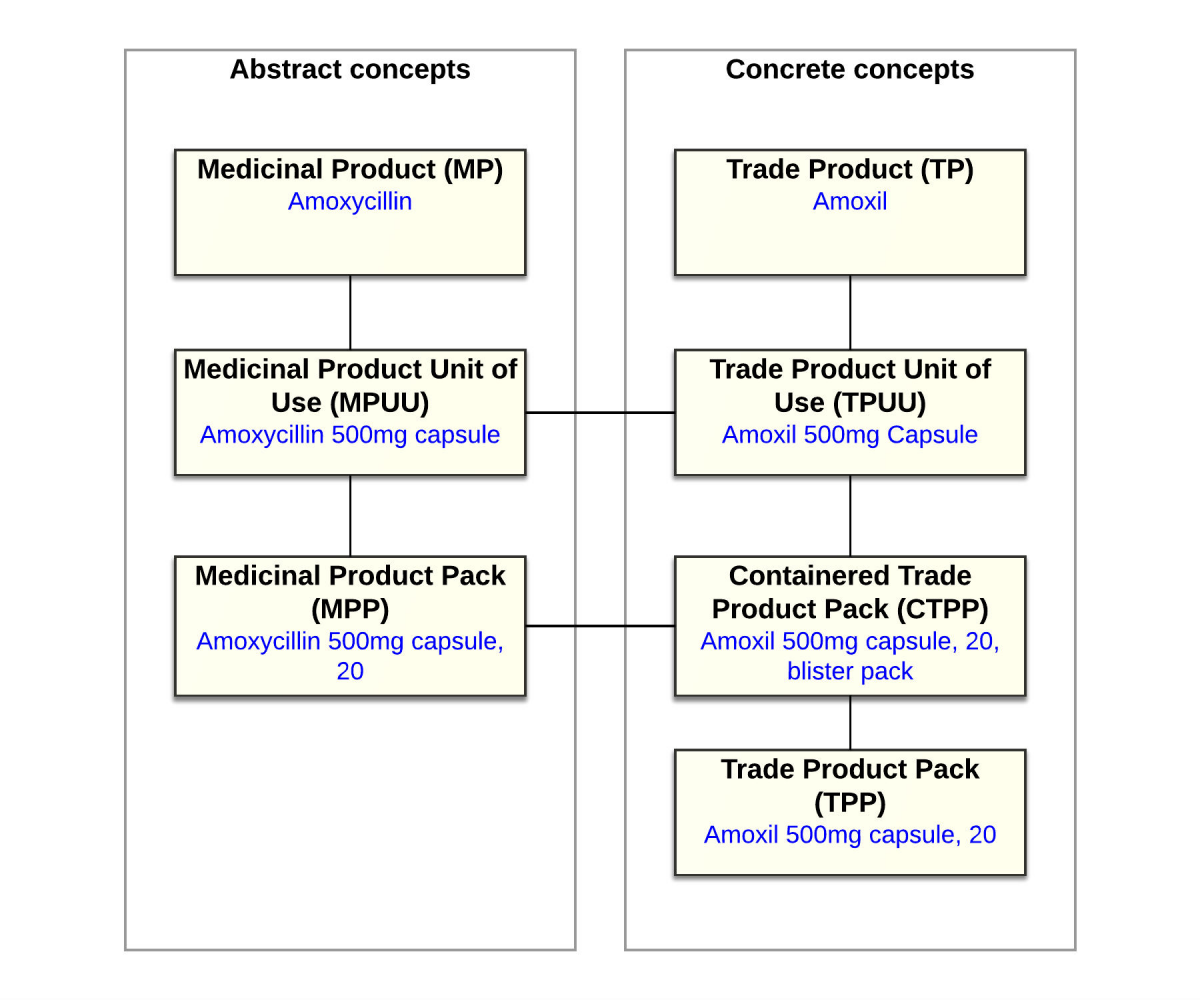
The hierarchical concepts used to describe medicines in the Australian Medicines Terminology.

In Australia, the Australian Digital Health Agency (ADHA) has created the Australian Medicines Terminology (AMT), an organized collection of codes and descriptions for uniquely identifying originator and generic medication brands used in Australia. The codes are hierarchically linked (see Figure 1) so that for any given medication it is possible to identify the active ingredients, dosage form, strength, trade name, pack type, and pack size [30]. By providing independent, unique codes, the AMT is intended to allow long-term, reliable communication of medication information between different systems. Uptake of the AMT has been slow, particularly in commercial software, but it is being increasingly used in research and analysis [31-33]. The AMT is a subset of the Systemised Nomenclature of Medicine Clinical Terms (SNOMED CT), an international standard for encoding clinical terms [34]. AMT equivalent systems exist outside of Australia, such as the UK NHS Dictionary of Medicines and Devices (dm+d) [35] or the Singapore Drug Dictionary (SDD) [36]. In the United States, drugs can be encoded using the US edition of SNOMED CT’s Product and Substance concepts or using the terminology set RxNorm, which has been mapped to SNOMED CT [37].

Given that collecting data on sedative use directly from nursing homes is impractical, pharmacy medication packing systems provide both the next-closest data point, plus the least number of data sources. They also provide the most consistently electronic records, including those kept within nursing homes. An ETL approach to retrieving data from pharmacy systems, combined with a validation and verification process from nursing staff at the point of care, was developed to provide accurate data to facilitate audits of sedative medication use, reporting and education, and personalized medication review plans for nursing home residents on a national scale.

## Methods

### Data Extraction

To collect medication data from community pharmacies, a website and client program was developed. Extracted data was securely sent to a webserver, which kept detailed transaction logs of every record added, modified, and deleted. The client program was able to extract data from the FRED, Webstercare, MPS [38], and Minfos [39] medication packing systems. Packing systems were prioritized over dispensing as they provide a simpler measure of quantities supplied. As per the requirements of the broader intervention, data was extracted as a “point-in-time” snapshot, and as with the previous RedUSe study, 3 snapshots were taken at 3-month intervals.

The collection of data is a process run by a staff member of the supplying pharmacy. The staff member

1. Creates an account on the website.
2. Logs into the website from a computer in the pharmacy and downloads and runs client.
3. In the RedUSe client software, follows prompts to identify pharmacy and packing software brand
  - For Fred and Webstercare, the software locates the packing database; if atypical, it asks staff for location or to select from multiple possibilities.
  - For MPS and Minfos, the staff member generates a medication report and opens it in the RedUSe client.
4. In the RedUSe client software, links the pharmacy system wing or home group names corresponding to the wing or home names of the RedUSe-participating nursing home(s).

Once the data source was identified in step 3, the software established a connection appropriate to the packing system (eg, opening a database connection or opening the file for reading). Resident and medication data for the chosen homes were then retrieved, with resident names encrypted. Medication data included a packing system medication identifier, medication name, instructions for use, quantity supplied, date started, date ceased, and prescriber. All data was compressed and transmitted to the server using transport layer security (TLS) encryption. The uploaded data from one extraction (referred to as “an upload”) contained all residents and medications for all currently participating nursing homes supplied by that pharmacy. The client program was a small (200 kilobyte) download.

### Data Enrichment

**Figure 2:**
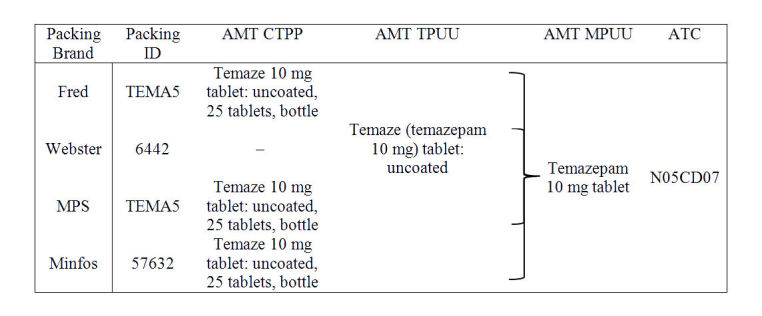
Example mappings for each packing software brand of a temazepam product.

The medication identifiers used by each of the packing systems were mapped to the appropriate AMT concept. Depending on the software, this was either the Trade Product Unit of Use (TPUU) or the Containered Trade Product Pack (CTPP). Using the AMT, these concepts were then translated to the corresponding Medicinal Product Unit of Use (MPUU). Finally, mappings were created between relevant AMT MPUU concepts and the appropriate ATC codes, providing a link from the medicine identifiers used in each packing program to a consistent naming scheme and drug classification system (see Figure 2 for examples). Where possible, mappings were created automatically based on known codes such as the Australian Register of Therapeutic Goods. Any missing mappings were added by a team of two research pharmacists.

Using the ATC code, medications of interest (for the RedUSe project, sedative medications) were automatically identified and aggregated based on the ATC codes shown in Table 1. Using this data, reports were generated by the server, on request, describing prevalence of antipsychotic and benzodiazepine medication use in each nursing home. Additionally, a measure of sedative load was calculated for each resident: the average daily dose for each medication was determined and compared with the World Health Organization’s Defined Daily Dose (DDD) values [40]; to enable reporting at a nursing home level, daily doses were converted to an equivalent strength of diazepam for benzodiazepines and chlorpromazine for antipsychotics. The calculations did not include sedative medications taken pro re nata (PRN, “as required”), rather than taken regularly, due to difficulty quantifying administered PRN doses.

It is noteworthy that this process was implemented using version 2 of the AMT; version 3 was released while this project was ongoing, but there was no capacity to convert while the project was running. As AMT version 2 did not include some newer medications, there were some medications that could not be automatically linked. The impact of this is discussed later.

**Table 1:** ATC codes used to identify drugs of interest.

ATC Code	Excluding	Name
N05A		Antipsychotics
	N05AN	Excluding Lithium
	N05AB04	Excluding Prochlorperazine
N05BA		Anxiolytics (Benzodiazepine derivatives)
N05CD		Hypnotics and Sedatives (Benzodiazepine derivatives)
N05CF		Benzodiazepine-related drugs
N03AE01		Clonazepam

### Data Validation and Verification

Once the medication data was uploaded, there was a series of validation and verification steps performed. First, the names of all medication records that were unable to be automatically linked to the AMT were sent to a research pharmacist. If the pharmacist identified any unlinked sedative medications, those mappings were added; if mappings could not be made (eg, the medication did not exist in the AMT), the medication was instead mapped to a chemically equivalent medication to ensure that all sedative use was captured in the resident’s DDD. The medication name used in the prescription data was still presented to nursing staff to avoid any confusion.

Following this, the uploaded data was checked by project staff for any obvious errors that would require the upload to be reattempted and for any clear mistakes that could be unambiguously corrected.

Next, the champion nurse at the nursing home was asked to log into the website and verify that all residents were present and that all sedative medications were present and correct (including name, dose, and instructions). Nurses were not asked to quantify administered doses of PRN medication. They were also asked to add any missing residents and medications, particularly looking for medication dosage forms that may have been packed separately (eg, drops, wafers, or injections). Separately packed medicines were sometimes not uploaded depending on the type of software used by the pharmacy and how the pharmacist recorded the medications. The champion nurse also removed any residents or medications that were incorrect or no longer current; if a resident was identified as no longer residing in the home, their medications were automatically excluded. For these checks, any missing or suspect data (such as doses falling outside of a typical range) were highlighted on the website. Once all residents had their sedative records verified, the option to generate the audit report and sedative review plans became available, with a review plan generated for every resident prescribed one or more sedative medications (the content and process for these review plans are beyond the scope of this paper).

**Figure 3:**
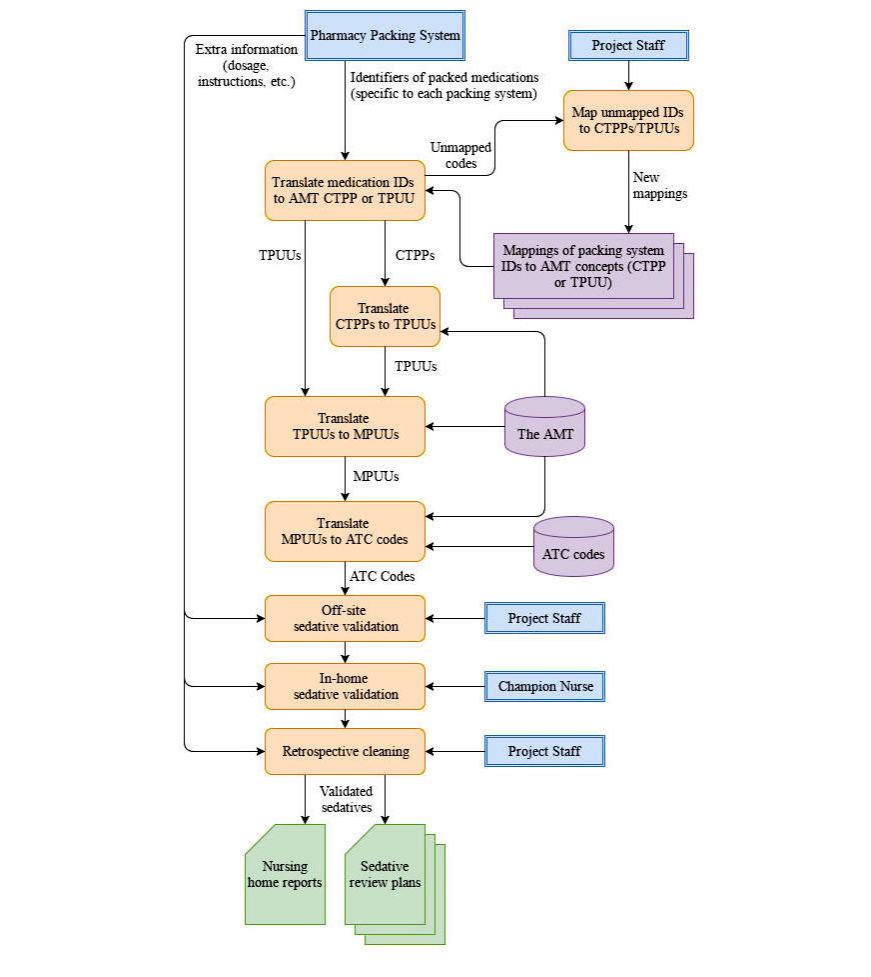
Extract, transform, and load (ETL) and validation process.

Final data validation was also performed retrospectively by project staff, primarily to merge duplicate or split resident accounts but also to improve the consistency of the data for final reports issued to nursing homes. This addressed issues introduced by errors in the extraction process and any inconsistencies in the previous validation steps between nursing homes. The full ETL and validation process is shown in Figure 3.

### Manual Data Entry

The typical approach to conducting medication audits in nursing homes is to visit the home and inspect the resident charts, recording the data by hand. If automated extraction of medication data was not possible, the champion nurse and regional project pharmacist used this as a fallback solution. In this case, the website functionality for data validation and verification could be used as an entry-assistance tool: forms for entering missing residents and medications already existed including auto-completing fields, instruction translation, and error highlighting. Using the same forms also ensured that entered medicines were in the same enriched format as uploaded medicines. To simplify this process, only sedative medication was required (defined according to Table 1 ATC codes), and any data entry snapshots after the first had the capacity to prepopulate from existing data, requiring only the changes since the last snapshot to be entered.

### Recruitment, Privacy, and Consent

Recruitment of nursing homes aimed to provide a range of sizes, locations, ruralities, and organizations. Two national nursing home groups offered all of their facilities to be involved. Two aged care advocacy bodies assisted in the recruitment of smaller organizations and independent nursing homes by featuring “RedUSe” in industry journals, resulting in over 300 expressions of interest. From this cohort, facilities were selected for invitation, with an effort to meet the described criteria. Invitations continued until consent was gained from the target number of 150 RACFs. Facilities with less than 29 residents or from outer rural or remote locations, and those located in the Northern Territory, were excluded for logistical reasons. Supply pharmacies were approached for consent once the nursing home agreed to participate. Pharmacies were provided detail on the project and the software and were offered a small remuneration for facilitating data collection for a nursing home.

During the project, identifiable patient information was encrypted on the server and only decrypted for authorized staff at that nursing home and the project staff member responsible for contact with that nursing home. Nursing home level aggregations were only available to the management of that home, the home’s pharmacist, the project staff member in contact, and the project lead.

Full ethical approval for the study was obtained from the Human Research Ethics Committee (Tasmania) Network (reference, H0013545).

## Results

### Extracted Data

Of participating nursing homes, 94.7% (142/150) had resident and medication data successfully uploaded via the automated process, supplied by 81 pharmacies. The eight participating nursing homes that did not have data uploaded were serviced by five pharmacies. One pharmacy owner declined to participate. The other four pharmacies used packing systems not supported by the extraction software: three used a platform that did not allow the software to be run; the other used a new brand of packing software.

Of the 150 nursing homes, 95.3% (143/150) were supplied by a single pharmacy, 4% (6/150) were supplied by two pharmacies, and one home was supplied by three pharmacies. On average, there were 90 residents per nursing home (standard deviation [SD]=37.5).

In total, 311 uploads were completed. Of these, 73.3% (228/311) included one nursing home, 21.9%, (68/311) included two nursing homes, 9 included three nursing homes, 5 included four nursing homes, and 1 included five nursing homes. Facilitating these took the supplying pharmacist a mean of 15 min per upload (standard error=0.67). Data upload size was, on average, 54 kilobytes.

**Figure 4:**
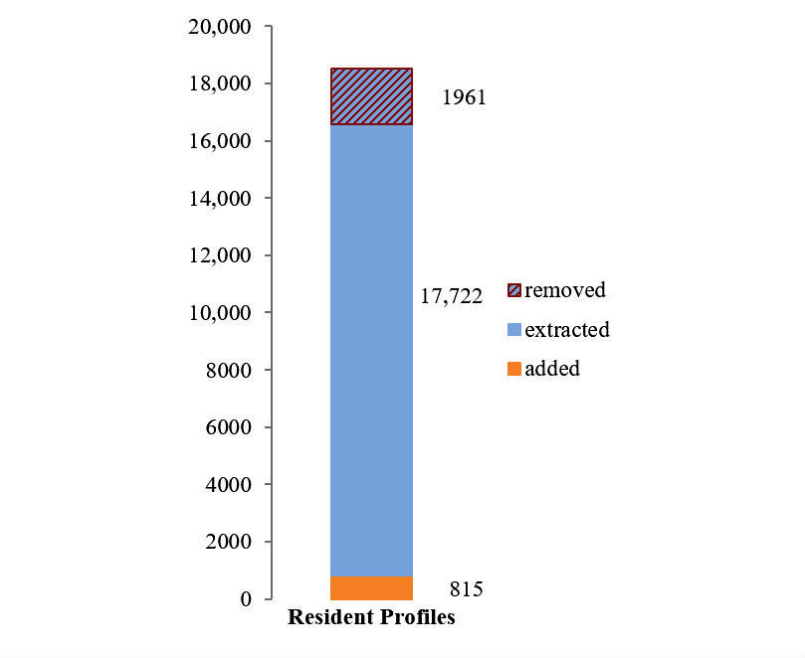
Residents extracted, removed, and added.

**Figure 5:**
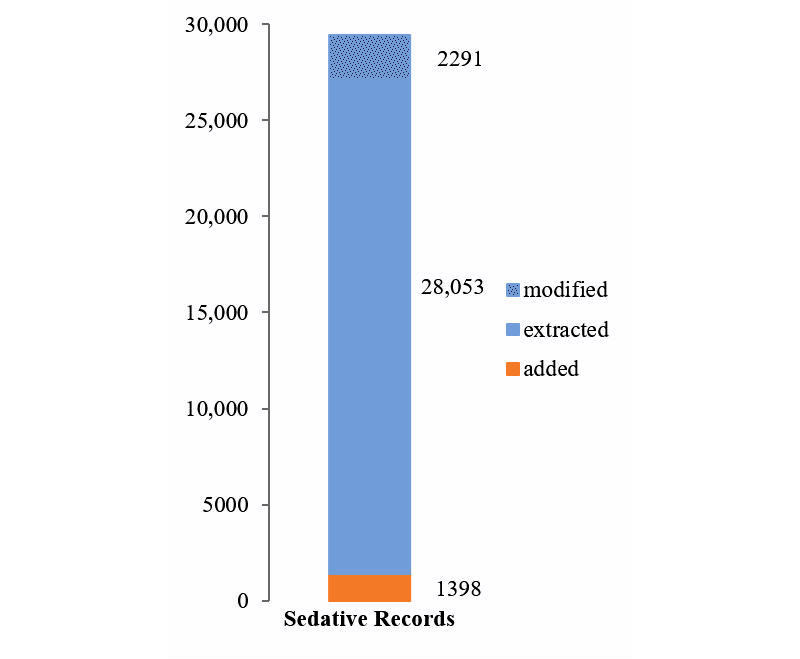
Sedatives extracted, corrected, and added.

Overall, 17,722 resident profiles were extracted, with 11.07% (1961/17,722) identified as duplicate or secondary records for an existing resident and merged into other records. For homes where data was automatically uploaded, 815 additional residents, or 4.4% of total (815/18,537) were added manually during data validation (shown in Figure 4). For uploaded residents, 546,535 medication records were extracted, of which, 5.13% (28,053/546,535) were identified as antipsychotics or benzodiazepines. There were 2291 antipsychotic and benzodiazepine records (8.16% of 28,053) modified during the validation steps, with a total of 3814 modifications. A further 4.75% (1398/29,451) of total antipsychotic and benzodiazepine medications were added manually (shown in Figure 5). Sedative medications taken PRN were significantly more likely not to be included in packing programs (3.62% [586/16,179] of regular medications were missed, against 6.12% [812/13,272] of PRN medications missed; two-tailed z-score −10.02, *P*<.001).

### Data Errors and Corrections

#### Validation and Verification

All manual corrections have been broken down into each validation and verification step in Table 2. In phase one, 9.56% (219/2291) of incorrect medications required a manual mapping. Of these, 82.2% (180/219) were recently introduced medications that would have been automatically linked if AMT version 3 was used; the remainder were due to out-of-date pharmacy vendor databases, unusual prescriptions (eg, brandy), or customized medications.

During the third phase validation, 48.69% (1870/3841) of all corrections were made by champion nurses and regional project pharmacists. Off-site validation and retrospective data cleaning, performed by project researchers, accounted for the remaining 44.91% (1725/3841) of modifications made.

#### Known Errors

Of the errors in extracted medicine data, the most frequent problems were missing dosage quantities. These could all be accounted for by three mistakes in the extraction software. One flaw, for one pharmacy software type, resulted in 325 records missing dosage numbers (24.83% (325/1309) of extracted records for that software in that time). A similar problem with another packing system accounted for another 284 records missing dosage numbers, or 8.73% (284/3254). Once corrected, subsequent dosage extractions from those software types required only 15 modifications, or 1.43% (15/1047) of records from that software in that time. The largest single cause of missing dosages was triggered by a pharmacy packing system changing their data structure during the project. This resulted in another 2108 records missing dosage numbers that needed to be entered based on the extracted instructions (representing 42.95% [2108/4908] of records in that time, up from 3.84% [125/3254] before the change). Altogether, these three missing dosage data issues accounted for 75.58% (2717/3595) of the sedative record corrections after mappings had been made. Removing these from consideration (as they are now corrected issues) alters the accuracy and validation requirements substantially, as shown in the second section of Table 2. Besides dosage quantities, all other extracted medicine attributes had approximately equal frequencies of error, with an average of 2% of uploaded records needing modification.

**Table 2:** Medication corrections made in each data validation and verification phase.

Phase	Corrections n (%)	Corrections excluding known errors n (%)
1: Identifying unmapped medications	219 (5.74)	219 (19.96)
2: Off-site validation	764 (20.03)	201 (18.32)
3: In-home verification	1870 (49.02)	384 (35)
4: Retrospective cleaning	961 (25.2)	293 (26.71)
	3814	1097

#### Time Requirements

Website interaction logs provided more detail of the on-site verification of data. By aggregating continuous blocks of interactions, we estimate that the champion nurse required a mean of 33 min of website interaction to check uploaded data for one nursing home; taking into account the size of the homes, this corresponded to 22 seconds per resident (SD=14.8). For homes where an automated upload was not possible, manually entering residents and their medications through the supplied RedUSe website required a mean of 60 min for the first snapshot, 43 min for the second snapshot, and 35 min for the third (shown in Table 3). This equated to 42 seconds per resident at baseline (SD=26.4) and 30 seconds per resident, subsequently (SD=15.2).

**Table 3:** The number and mean min required to perform data validation, for uploaded versus manually entered data.

Data source	Snapshot	Mean minutes for data entry (Initial validation and on-site verification)	Number of snapshots validated
Manual			
	baseline	60.0	20
	3 months	43.2	13
	6 months	35.2	13
	all manual	48.0	46
Uploaded			
	baseline	38.1	130
	3 months	31.3	137
	6 months	31.3	137
	all uploaded	33.5	404

## Discussion

These results demonstrate a successful implementation of an ETL method for retrieving and verifying sedative medication prescribing in nursing homes, on a national scale. This section explores the extent of that success and discusses considerations for future applications of the method.

### Upload Coverage

The data extraction method was generally applicable across pharmacies. Apart from one that declined to participate, there were only four pharmacies (5% of the 86 pharmacies invited) that were unable to have medication data uploaded. These were due to two uncommon packing systems, both of which can be included with further development of the extraction software. Likewise, most residents (95.6%, 17,722/18,537) and most of their sedative medications (95.26%, 28,053/29,451) were included in uploads. The residents that were missed were due to individuals being supplied by an alternate pharmacy or due to new residents entering the home between the time the upload occurred and the time the data validation was performed. Similarly, medications could be missed due to this time difference but also due to differences in pharmacy packing protocols: for example, 6.12% (812/13,272) of PRN medications were not included in packing, requiring identification and manual addition during data validation. In addition, certain medication forms presented problems for extraction. For one packing system, injections, wafers, and solutions were always missed; for another, their inclusion was heavily dependent on the particular pharmacy’s recording and packing practices, with approximately 1 in 5 pharmacy uploads not including those medications. These highlight the value of the manual validation steps; for example, if timeliness of data is an issue, or if 95% coverage of PRN medications is insufficient, then manual validation is a necessity.

### Data Accuracy

Nearly 92% (25,762/28,053) of sedative medications were correct as uploaded. This rate improved to 98.32% (27,583/28,053) if the “known” errors in extraction, including the outdated AMT, were excluded. Given that those errors were corrected, it is tempting to expect that any future use of this data extraction method would achieve that rate, but this may be unrealistic: the major “known” error was caused by an external update to pharmacy software, and the frequency and timing of those updates is unpredictable.

Other medication data errors were due to individualities in pharmacy packing systems. In the initial validation phase, 82.2% (180/219) of the unknown medication errors were due to using an outdated version of the AMT. The other 17.8% were caused by pharmacy-unique entries: packing systems allow pharmacists to add medications, for new products released before the system vendor can update their database or for pharmacy-prepared medications. Errors in recognized medications were caused by inconsistent entry into the packing systems, such as incomplete records, transposed fields, or typing mistakes. Retrospective corrections were primarily to merge multiple resident profiles: residents were often entered into pharmacy systems more than once, either as an oversight or intentionally to split medication packs. Other retrospective medication data changes were made for internal consistency (eg, correcting dosage quantities to match those given in the instructions).

That 25.2% (961/3814) of the corrections were performed retrospectively might seem to cast doubt on the accuracy of the previous validation steps. It is possible that some mistakes were made but unlikely, given the extensive validation process where the champion nurse, pharmacist, and research team all reviewed the data.

The volume of errors corrected highlights the need for data validation and verification. Adaptability of the extraction software is expected to be crucial for any longitudinal use of this approach as packing systems change between pharmacies and over time. Verifying uploaded data is a crucial step in identifying when those changes occur. Given the accuracy achieved within this project, if due consideration is given to validation, verification, and maintenance, it may be reasonable to aim for an accuracy rate above 95% (before corrections).

### Comparison With Manual Entry

Compared with the automated process, the time recorded for manual data entry does not appear too unfavorable, especially when considering the additional 15 min required for the community pharmacist to facilitate an upload; however, this “manual” process was facilitated by a project-developed website with entry-assistance, so it does not fully describe the time benefit of the automated process. The prepopulation of data explains most of the increase in efficiency between the baseline and 3-month snapshots shown in Table 3; the remainder is due to familiarity with the website. It is also notable that although this application was only interested in sedative medications, the automated upload included all medications supplied by the pharmacy, and manual entry only included sedative medications. This translated to an average of 10 more medications per resident (11.84 per uploaded resident against 1.96 per manually added resident).

### Applicability of Results

Although this study only validated sedative data, similar accuracy is expected for other medication classes, and the validation cost would be expected to increase proportionally. Maintenance costs should be approximately equivalent; systemic changes typically require the same amount of work regardless of the classes of medication. Managing the burden on nursing staff should be a key consideration, potentially by utilizing more nurses or reducing other project responsibilities.

Although this study included four Australian pharmacy packing systems, the approach is independent of particular systems. The model of community pharmacies supplying medication to nursing homes appears to be the standard in the United States, the United Kingdom, and elsewhere. The complexity of implementing this approach will be dependent on the range of software used by those pharmacies, which will differ by region. Of more significance is the relative uptake of standardized EHRs in the wider health system. If pharmacy software encodes medications to a recognized standard (such as SNOMED CT), then the data transformation cost and maintenance cost of the extraction software is drastically reduced. A standardized, appropriately encoded, widely adopted, and accessible patient EHR would make this data collection approach unnecessary. While many countries are attempting to move toward such a system, the authors are not aware of any that yet meet these criteria.

### Conclusions

Extracting pharmacy medication packing records and enriching them through mapping to an independent medication data resource, such as the AMT and ATC codes, was used to collect sedative medication usage data in remote nursing homes on a national scale, with success in 94.6% (142/150) of the homes involved. Without data validation and verification, the accuracy rate for sedative medications could be expected to be above 90% and possibly over 95%. At least 20% of these errors would be expected to be unambiguously correctable with only basic validation. Although this unverified data may be unsuitable for individualized clinical applications, it is useful as a data source for more generalized analyses and avoiding the validation step makes it a much faster process.

With data validation and verification steps included, the method compares favorably to in-person visits and manual resident chart reproduction, taking less time and producing more data. It is also highly scalable. However, to produce reliable clinical data, continual monitoring of data accuracy is essential not only to correct mistakes in individual pharmacy data entry but also to identify variations in pharmacy packing systems. These variations also mean that there needs to be capacity for maintenance to the extraction software.

## Abbreviations

ADHA: Australian Digital Health Agency
AMT: Australian Medicines Terminology
ATC: Anatomical Therapeutic Chemical
BPSD: Behavioral and Psychological Symptoms of Dementia
CTPP: Containered Trade Product Pack
DDD: defined daily dose
dm+d: Dictionary of Medicines and Devices
ETL: extract, transform, and load
MPUU: Medicinal Product Unit of Use
PRN: pro re nata (“as needed”)
RedUSe: Reducing the Use of Sedatives project
SDD: Singapore Drug Dictionary
SNOMED CT: Systemized Nomenclature of Medicine Clinical Terms
TLS: transport layer security
TPUU: Trade Product Unit of Use
